# Mitochondrial genome analysis of the spoon-billed sandpiper (*Eurynorhynchus pygmeus*)

**DOI:** 10.1080/23802359.2017.1298415

**Published:** 2017-03-17

**Authors:** Hey-Sook Joen, Mu-Yeong Lee, Yu-Seong Choi, Junghwa An

**Affiliations:** a Animal Resources Division, National Institute of Biological Resources, Incheon, Republic of Korea;; b Division of Basic Research, National Institute of Ecology, Chungcheongnam-do, Republic of Korea

**Keywords:** *Eurynorhynchus pygmeus*, spoon-billed sandpiper, mitochondrial genome

## Abstract

The complete mitogenome sequence of Spoon-billed sandpiper (*Eurynorhynchus pygmeus*) was reported in this study. The mitogenome was a circular molecule (16,709 bp) with a typical vertebrate mitogenome arrangement, which consisted of 13 protein-coding genes, two ribosomal RNA genes, 22 transfer RNA genes, and one non-coding region (D-loop). The overall base composition was 31.3% A, 29.9% C, 13.8% G, and 25.0% T. The length of D-loop is 1155 bp in length, containing tandem repeats. Phylogenetic analyses based on the concatenated protein-coding genes indicated that the South Korean and Chinese *E. pygmeus* formed a group that was most closely related to *Arenaria interpres*.

The spoon-billed sandpiper *Eurynorhynchus pygmeus* (Aves, Charadriiformes, Scolopacidae) is a small shorebird with a characteristic broad-shaped beak. It breeds in the Chukotka and Koryakya regions in north-eastern Russia, migrates to Korea and Japan, and winters in south-eastern Asian countries such as Vietnam, Thailand, and Myanmar (Tomkovich et al. 2002; Zöckler et al. 2010). This species is listed as Critically Endangered in the International Union for the Conservation of Nature (IUCN 2016) Red List because of its habitat loss, caused by composite factors including habitat disturbance, pollution, hunting, and the effects of climate change (BirdLife International 2017). In South Korea, the most threatening factor to the spoon-billed sandpiper is the reduction of food resources due to mud flat reclamation. The Ministry of Environment of Korea classified this species as an endangered species I (National Institute of Biological Resources 2011).

In the present study, we sequenced and characterized the mitogenome of *E. pygmeus*. A sample (IN316) of *E*. *pygmeus* was collected in Yubu Island, Seocheon-gun, Chungcheongnam-do, South Korea, and deposited in the National Institute of Biological Resources at Inchoen, South Korea. Total genomic DNA was extracted from dried blood using the QIAamp^®^ DNA Micro Kit (Qiagen Inc., Valencia, CA) and genome annotation was performed using DOGMA (Wyman et al. 2004) and ARWEN (Laslett & Canbäck 2008). The complete mitogenome of *E*. *pygmeus* was 16,709 bp in length (GenBank accession No. KY434065) and its overall base composition included 31.3% A, 29.9% C, 13.8% G, and 25.0% T. The complete mitogenome arrangement of *E*. *pygmeus* was similar to that of other birds and vertebrates (Sorenson et al. 1999), including 13 protein-coding genes, 22 transfer RNA genes, two ribosomal RNA genes, and one control region (D-loop). The D-loop (1155 bp) contained tandem repeats consisting of AAAC. As recently reported for species in Charadriiformes such as *Haematopus ostralegus* and *Charadrius placidus* (Lee et al. 2017a, 2017b), the mitogenome of *E*. *pygmeus* contained a frameshift mutation in *ND3*, corresponding to a “C” in position 174. Mindell et al. (1998) also illustrated this phenomenon in some birds and turtles.

For the phylogenetic analysis, we selected the 13 protein-coding genes (11,398 bp) of *E. pygmeus* from South Korea, the Chinese *E. pygmeus* (Accession No. KP742478), and four other species within Scolopacidae. The neighbour-joining tree constructed in MEGA6 (Tamura et al. 2013) showed that the South Korean and Chinese *E. pygmeus* formed a group (Figure 1) that was most closely related to *Arenaria interpres*. This result was similar to that presented in Ge et al. (2015). The complete mitogenome of *E. pygmeus* presented here conveys important data for molecular species identification and to further assess phylogenetic relationships within Scolopacidae.

**Figure 1.**
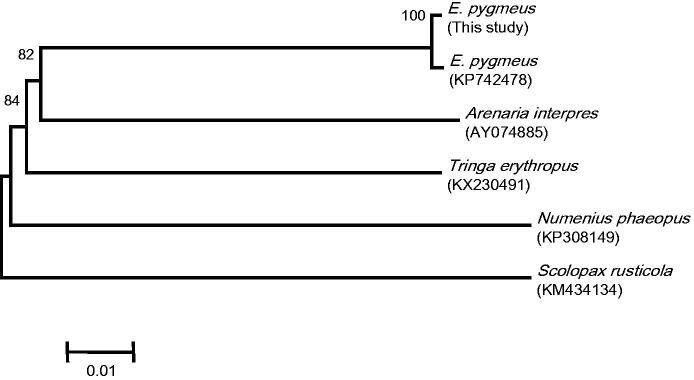
The neighbour-joining phylogenetic tree of spoon-billed sandpiper and four species of family Scolopacidae based on the concatenated nucleotide sequences of 13 protein-coding genes. Bootstrap replicates were performed 1000 times.

## References

[CIT0001] BirdLife International 2017 Species factsheet: *Calidris pygmaea* Downloaded from http://www.birdlife.org on 02/02/2017.

[CIT0002] Ge X, Hu D, Zhao F, Yu T, Zhang B, Chang Q. 2015 The complete mitochondrial genome of *Eurynorhynchus pygmeus* (Charadriiformes: Scolopacidae). Mitochondrial DNA A. 27:2473–2474. 10.3109/19401736.2015.1033703 26065849

[CIT0003] Laslett D, Canbäck B. 2008 ARWEN: a program to detect tRNA genes in metazoan mitochondrial nucleotide sequences. Bioinformatics. 24:172–175. 18033792 10.1093/bioinformatics/btm573

[CIT0010] Lee MY, Jeon HS, Choi YS, Joo SB, An JH. 2017a Complete mitochondrial genome of *Haematopus ostralegus* (Charadriiformes: Haematopodidae). Mitochondrial DNA B. 2:124–125. 10.1080/23802359.2017.1292474 PMC7799934 33473739

[CIT0011] Lee MY, Jeon HS, Lee SH, An JH. 2017b The mitochondrial genome of the long-billed plover, *Charadrius placidus* (Charadriiformes: Charadriidae). Mitochondrial DNA B. 2:122–123. 10.1080/23802359.2017.1292473 PMC7800784 33473738

[CIT0012] Mindell DP, Sorenson MD, Dimcheff DE. 1998 An extra nucleotide is not translated in mitochondrial ND3 of some birds and turtles. Mol Biol Evol. 15:1568–1571. 12572620 10.1093/oxfordjournals.molbev.a025884

[CIT0004] National Institute of Biological Resources. 2011 Red Data Book of Endangered Birds in Korea. Incheon, South Korea: NIBR.

[CIT0005] Sorenson MD, Asr JC, Dimcheff DE, Yuri T, Mindell D. 1999 Primers for a PCR-based approach to mitochondrial genome sequencing in birds and other vertebrates. Mol Phylogenet Evol. 12:105–114. 10381314 10.1006/mpev.1998.0602

[CIT0006] Tamura K, Stecher G, Peterson D, Filipski A, Kumar S. 2013 MEGA6: Molecular evolutionary genetics analysis version 6.0. Mol Biol Evol. 30:2725–2729. 24132122 10.1093/molbev/mst197 PMC3840312

[CIT0007] Tomkovich PS, Syroechkovski EE Jr, Lappo EG, Zöckler C. 2002 First indications of a sharp population decline in the globally threatened Spoon-billed Sandpiper *Eurynorhynchus pygmeus*. Bird Conserv Int. 12:1–18.

[CIT0008] Wyman SK, Jansen RK, Boore JL. 2004 Automatic annotation of organellar genomes with DOGMA. Bioinformatics. 20:3252–3255. 15180927 10.1093/bioinformatics/bth352

[CIT0009] Zöckler C, Syroechkovskiy EE, Atkinson PW. 2010 Rapid and continued population decline in the Spoon-billed Sandpiper *Eurynorhynchus pygmeus* indicates imminent extinction unless conservation action is taken. Bird Conserv Int. 20:95–111.

